# Storm-Induced Wind Damage to Urban Trees and Residents’ Perceptions: Quantifying Species and Placement to Change Best Practices

**DOI:** 10.3390/plants14213366

**Published:** 2025-11-03

**Authors:** Attila Molnár V., Szabolcs Kis, Henrietta Bak, Timea Nagy, Attila Takács, Mark C. Mainwaring, Jenő Nagy

**Affiliations:** 1 HUN-REN–UD Conservation Biology Research Group, 4032 Debrecen, Hungary; kis.szabi17@gmail.com (S.K.); henriettabak17@gmail.com (H.B.); tima.nagy@gmail.com (T.N.); limodorum.abortivum@gmail.com (A.T.); jenonagy.off@gmail.com (J.N.); 2 Department of Botany, Faculty of Science, University of Debrecen, Egyetem Sq. 1, 4032 Debrecen, Hungary; 3 Botanical Garden, University of Debrecen, Egyetem Sq. 1, 4032 Debrecen, Hungary; 4 School of Environmental and Natural Sciences, Bangor University, Bangor LL57 2DG, UK; m.mainwaring@bangor.ac.uk

**Keywords:** climate change impacts, damage assessment, green infrastructure, urban forestry management, urban green space protection

## Abstract

Tree-covered urban green spaces, including streets, parks, and other public areas, are vital for urban sustainability and people’s well-being. However, such trees face threats from the occurrence of extreme weather. In this study, we investigated wind damage to urban trees in the city of Debrecen, Hungary, during two severe windstorms in July 2025. Field surveys were conducted across three distinct urban zones, covering approximately 515,000 m^2^ in total. We assessed 201 damaged and 325 undamaged trees and recorded the species, size, damage type, and contextual landscape features associated with them being damaged or not. Damage type to trees consisted primarily of broken branches, whilst uprooting and trunk breakage were recorded less often. Most tree characteristics (trunk circumference, height, systematic position, nativity) and the proximity and height of buildings upwind of focal trees were significant predictors of their vulnerability to windstorms. In addition, we surveyed 150 residents in person and received comments from 54 people via online questionnaires and explored their perceptions of storm frequency, the causes of storms, and mitigation measures. Most respondents noted increased storm frequency and attributed that to climate change, and they suggested mitigation measures focused on urban tree management and environmental protection. Some people expressed scepticism about the presence of climate change and/or their ability to address such damage on an individual basis. Our study is the first to integrate assessments of storm-related impacts on urban trees with the opinions of residents living in proximity to them. Our findings highlight the need for climate-adaptive and mechanically robust urban forestry planning and offer insights that guide the management of trees in urban areas globally. Specifically, we propose to undertake the following: (1) Prioritise structurally resilient, stress-tolerant tree species adapted to extreme weather conditions when planting new trees. (2) Integrate wind dynamics, microclimatic effects and artificial stabilisation techniques into urban design processes to optimise tree placement and their long-term stability. Urban planners, builders, developers, and homeowners should be informed about these stabilising practices and incorporate the needs of trees early in the design process, rather than as decorative additions. (3) Develop regionally calibrated risk models and early-warning systems to support proactive and data-driven tree management and public safety. (4) Promote climate literacy and public participation to strengthen collective stewardship and resilience of urban trees.

## 1. Introduction

Trees adapt to strong winds through structural reconfiguration and the shedding of branches, yet particularly strong winds may cause damage to roots, thereby causing fatal damage. Strong winds also drive forest regeneration and succession, thereby highlighting the diverse effects of high wind speeds upon trees [1,2].

Tree-covered urban green spaces including streets and parks are indispensable features of sustainable urban areas that are beneficial to biodiversity and humans alike. However, the structural integrity of trees in urban and rural areas are being increasingly threatened by the occurrence of extreme weather events, including high-intensity windstorms, which frequently result in trees falling to the ground, infrastructural damage, and subsequent threats to human lives [1,2].

Numerous studies have sought to identify the factors that influence urban tree vulnerability during extreme weather events associated with high winds [3,4,5]. The relationship between tree architecture and the risk of them falling is well-documented: for instance, interspecific differences in storm susceptibility were highlighted in the city of Guangzhou, China, with *Ficus* species proving to be particularly vulnerable to extreme weather events [6]. The risk of wind damage to trees is determined by a range of interacting factors, including wind characteristics, species-specific tree traits, and the structures of stands of trees such as the density of tree trunks [2,7]. A wind fragility model based on the mechanical analysis of urban street trees helped identify which trees were susceptible to being damaged, and possibly falling, during extreme windstorms [8].

Beyond intrinsic tree characteristics, canopy density, soil moisture, and rainfall–wind interactions, which influence the drag of trees and thereby their anchorage strength, play a key role in determining tree stability and susceptibility to windstorms [2,9,10,11]. One study reported a 30% increase in the probability of trees being uprooted under saturated soil conditions during high rainfall [12], highlighting the compounding effect of precipitation and wind on tree damage [13]. Meanwhile, longitudinal data from Venice, Austria, showed that wind gusts exceeding 15 m/s, in conjunction with accumulated rainfall, substantially increased the rates of trees falling to the ground [14].

Urban structures, such as buildings, strongly determine wind exposure risk to individual trees. Illustratively, a “wind risk index” developed for the city of Milan, Italy, identified exposed street corridors and isolated trees as being locations in which trees were susceptible to high winds [15]. Data from Venice revealed spatial clustering of trees falling to the ground near impervious surfaces and open intersections where roots were unable to become established and thereby support the trees [14]. Emerging approaches, including fragility curve modelling, computational fluid dynamics, and machine learning, have allowed for more nuanced risk assessments to be performed at the scale of individual trees [7,8,16], and the feasibility of real-time early warning systems based on rainfall and gust forecasts can help forecast the fate of individual trees under severe weather events [2,12].

The probability of trees remaining undamaged during extreme weather events is therefore determined by a variety of factors, arising from the intersection of biomechanical traits, weather conditions, and the layout of buildings within urban areas [2]. Effective mitigation measures are therefore likely to include soil and tree canopy management, and informed urban planning that reduces the exposure of trees to extreme windstorms. Nevertheless, studies that examine the susceptibility of trees to extreme weather events remain sparse [8,15].

In Hungary, research on urban trees has primarily addressed the ecosystem services they provide, including carbon sequestration, dust and pollutant filtration, and environmental bioindication [17]. For instance, studies have quantified foliar traits and metal accumulation in relation to air pollution [17,18], while dendrochemical analysis has been used to reconstruct urban pollution histories in the city of Szeged [19]. The contribution of trees in streets and parks to carbon storage and air purification has also been quantified [20], thereby highlighting the value of green infrastructure. Meanwhile, a remote-sensing-based tree classification system was developed for the city of Debrecen to aid green network design [21], whilst other studies have evaluated the structural and visual performance of trees in the city of Budapest [22]. Despite these examples, we are aware of no studies that have examined the effect of extreme weather events, particularly in the form of windstorms, on trees in urban areas of Hungary.

**Figure 1:**
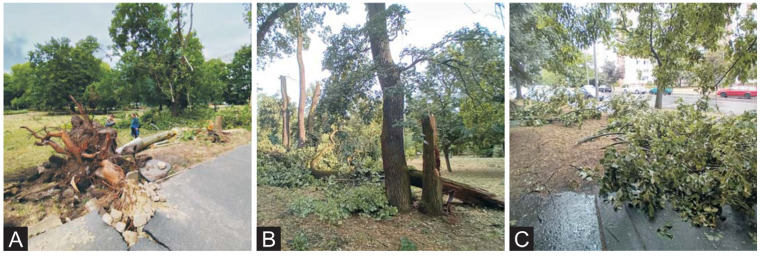
Types of storm damage on trees. (**A**)—*Platanus* × *hispanica* with a trunk circumference of 143 cm, uprooted at Böszörményi street. (**B**)—*Quercus robur* with broken trunk with 240 cm circumference in the campus of the University of Debrecen, (**C**)—broken branches of different species (mainly *Tilia argentea*) on Bolyai street.

The varied landscapes of Hungary, ranging from lowland plains to mountain regions, generate heterogeneity in local wind regimes, necessitating region-specific assessments of urban tree vulnerability. In large urban areas, where trees grow amongst buildings and other infrastructure, factors such as species adaptability, infrastructural interactions (e.g., soil compaction, rooting space), and local microclimates may significantly affect tree stability during and immediately after strong winds. The second most populous city (201,000 inhabitants) [23] of Hungary is Debrecen, situated in the north-eastern lowlands. On 7 July 2025 at 18:00 and again on 9 July at 15:00, the city experienced severe windstorms originating from the west and north-west directions. Peak wind gusts exceeded 130 km/h on 7 July, and reached approximately 111 km/h on 8 July, accompanied by 24 mm of rainfall [24]. The storm induced widespread tree damage, including trees being uprooted, twisting of trunks, branch breakage (Figure 1), and structural damage to adjacent infrastructure.

The objective of this study was to quantify the factors, namely tree species, nativity status, trunk circumference, height of canopy, and surrounding landscape characteristics, that contributed to the exposure and vulnerability of urban trees to storm damage within the city of Debrecen. In addition to the ecological and spatial aspects outlined above, the study also incorporates an assessment of residents’ perceptions regarding the frequency of storms, the causes of such storms, and mitigation measures [25]. Our findings can be used to help inform the tree species that fare best in urban areas, urban planning, and forestry practices that enable tree survival in a changing climate, while also contributing to a more inclusive understanding of societal awareness and attitudes towards climate-related disturbances to trees within the vicinity of their urban homes.

## 2. Results

### 2.1. Tree Damage and Species Composition

A total of 201 damaged and 325 undamaged trees were assessed for storm-induced wind damage. In all three surveyed areas, the most frequent type of damage was broken branches, affecting 31.0% of all recorded trees (81.1% of damaged trees). Meanwhile, uprooting (4.8%; 6.5% of damaged trees) and trunk breakage (2.5%; 12.4% of damaged trees) occurred much less frequently (Table 1), yet still represented common forms of damage to trees during windstorms.

A total of 70 tree species were identified in this study, of which 25 were native to Hungary and 11 were native to the city of Debrecen (Table 2). Among the tree species native to Debrecen, 48 undamaged individual trees from 9 species and 29 damaged individual trees from 6 species were recorded. Among the 59 species introduced to Debrecen, we surveyed 277 undamaged individual trees belonging to 48 species and 172 damaged individual trees belonging to 41 species. For the eight most common tree species (*Acer platanoides*, *Tilia tomentosa*, *Quercus robur*, *Robinia pseudoacacia*, *Acer saccharinum*, *Platanus* × *hispanica*, *Celtis occidentalis*, *Prunus cerasifera*), both damaged and undamaged individual trees were surveyed, totalling 289 individual trees. Together, these species accounted for 54.9% of all individual trees examined in the study.

The most frequently recorded type of damage to trees involved branch breakage, which was documented for 163 individual trees, resulting in a total of 284 broken branches (mean ± SD = 1.8 ± 0.9 per individual tree). Those broken branches had an average diameter of 10.8 ± 8.0 cm and an average length of 4.5 ± 2.5 m. The thickest broken branch had a diameter of 57 cm, while the longest branch reached 15 m, thereby indicating that large branches were broken during the windstorms.

The greatest risks to human health, buildings and other infrastructure, and vehicles were associated with trees that had been completely uprooted. A total of 25 uprooted trees were recorded, representing 15 tree species. The tree species most frequently found uprooted were *Abies cilicica* (4 individuals), *Acer saccharinum* (3), *Morus alba* (3), *Platanus* × *hispanica* (2), *Populus alba* (2), and *Pseudotsuga menziesii* (2). Their height ranged from 4 to 25 m, with an average of 13.9 ± 5.5 m, while the circumference of their trunks at breast height (CBH) ranged from 50 to 280 cm, with an average of 133.8 ± 62.9 cm. Notably, the proportion of gymnosperm evergreens was significantly higher among uprooted trees (40.0%) compared to both damaged trees overall (8.0%, Binomial test: *p* < 0.001) and the total sample of surveyed trees (12.9%, Binomial test: *p* < 0.001).

### 2.2. Factors Affecting Storm-Induced Wind Damage

**Figure 2:**
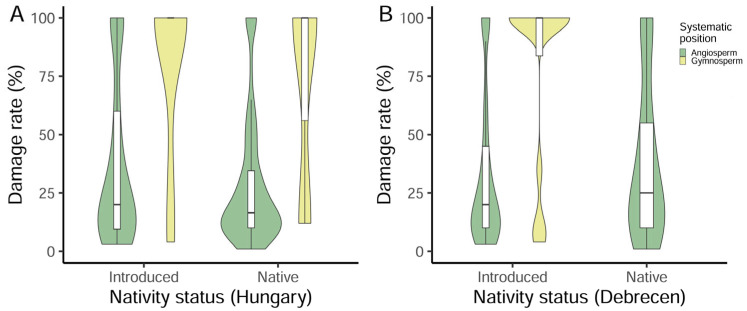
Summary plots for the estimated damage rate of trees (*n* = 201) grouped by nativity status in Hungary (**A**) and in Debrecen (**B**) in relation to their systematic position. Shapes indicate the distribution of values, and boxes and whiskers show the minimum, first quartile, median, third quartile and maximum values.

**Figure 3:**
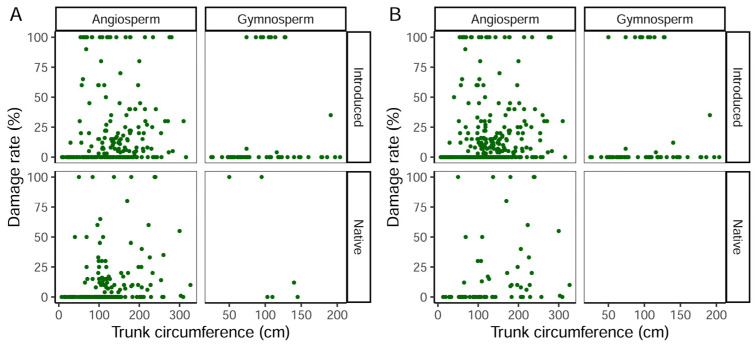
The relationship between the estimated damage rate and breast height trunk circumference of trees (*n* = 526) split by systematic position and nativity status in Hugary (**A**) and in Debrecen (**B**). Green dots represent individual trees.

The zero-inflated mixed-effect beta regression models indicated that both tree characteristics and surrounding landscape components influenced incidents of storm-damaged trees (Table 3). Gymnosperms were damaged significantly less often than angiosperms, yet when they were damaged by storms, the rate of damage they incurred was significantly higher than was observed in angiosperms. Tree species native to Hungary were more likely to remain undamaged during the storms (Figure 2A), but tree species native to Debrecen had higher rates of damage compared to introduced tree species (Figure 2B). Larger trees, as indicated by their CBH, had lower rates of damage from the windstorms (Figure 3A,B), whilst tree height was positively associated with the rate of storm-induced wind damage. The presence of buildings closer to focal trees, as well as higher buildings, both significantly decreased the probability of a tree being damaged during the storms.

### 2.3. Perceptions of Storm Frequency and Causes of Storms

#### 2.3.1. Perceived Changes in Storm Frequency

The majority of the 204 interviewees (143 respondents, 70%) believed that the frequency of storms had increased over recent decades. Meanwhile, 48 individuals (24%) perceived no change in the frequency of storms, while two respondents (1%) were unable to form an opinion. In contrast, eleven people (5%) believed that the frequency of storms had decreased in recent years. The age of interviewees who thought that the frequency of storms had increased over recent timeframes did not differ from the age of interviewees who did not think that the frequency of storms had increased (Wilcoxon’s test, W = 4609, *p* = 0.522).

#### 2.3.2. Perceived Causes of Storms

Respondents’ opinions regarding the causes of extreme storms were diverse. However, most people (104 individuals, 53%) invoked climate change or global change as a contributing factor. Additionally, six respondents (3%) invoked environmental pollution, and 47 respondents (23%) referred to weather events or extreme weather conditions. A further 13 participants (6%) attributed the causes to human impact, without explicitly mentioning climate change. One respondent (<1%) explicitly stated that, in their view, climate change was not responsible. Meanwhile, three participants (1%) mentioned conspiracy theories, claiming that politicians and atmospheric scientists were deliberately inducing adverse environmental changes, whilst two respondents (1%) referred to God as a causal factor. Finally, 19 individuals (9%) either did not know or chose not to answer the question. Interviewees who thought that climate change was the ultimate cause for extreme storms were significantly younger than those who did not think that climate change was the cause of extreme storms (Wilcoxon’s test, W = 3969, *p* = 0.003).

#### 2.3.3. Suggested Responses and Coping Strategies

In response to the question “How can we protect ourselves from storms and mitigate their impacts?”, most participants suggested symptomatic or localised interventions. A total of 23 individuals (11%) recommended the regular pruning of urban tree branches, whilst 58 (28%) individuals emphasised the importance of monitoring and maintaining tree health. Additional suggestions included: “there are too many trees, and they are too old”, “parking under trees should be prohibited”, and “urban trees should not be cut down”. Meanwhile, nine individuals (4%) proposed planting storm-resistant tree species or evergreen trees.

Some respondents reflected on broader systemic solutions. Eleven (5%) individuals highlighted the importance of environmental protection and reducing pollutant emissions, whilst two (1%) individuals mentioned the use of renewable energy sources, and eight people (4%) emphasised the need for a more conscious lifestyle. 34 respondents (17%) indicated the need for modifying tree planting policies or infrastructure to protect trees. Another two individuals (1%) advocated for greater attentiveness to both nature and human well-being. Conversely, however, 21 (10%) individuals believed that people, particularly individuals, are powerless to take meaningful action, and 31 individuals (15%) were unable to provide an answer.

## 3. Discussion

The effect of extreme windstorms in the city of Debrecen highlighted the increasing vulnerability of urban trees to extreme weather events. Our results revealed that storm-induced wind damage to urban trees was determined by a variety of biological, structural, and environmental factors, consistent with findings from previous studies of urban trees in temperate [7] and Mediterranean [7,14,15] climates. The predominance of branch breakage (81% of damaged trees) during such extreme weather events corroborates earlier observations that branch breakage is the most common form of damage to trees under moderate and strong winds [2]. Meanwhile, the uprooting of trees and trunk breakage occur less frequently than branch breakage yet pose a greater risk to public safety and infrastructure [14,15].

Tree-specific characteristics such as their height, trunk circumference, nativity, and systematic position emerged as significant predictors of damage severity during windstorms. Notably, taller trees were more susceptible to storm-induced wind damage, likely due to their increased exposure to windstorms and drag force at greater heights [2,12]. In contrast, trees with larger trunk circumferences experienced reduced damage rates during storms, possibly reflecting age-related structural robustness or deeper root systems [12]. The higher vulnerability of gymnosperm evergreens, particularly in terms of being uprooted, are likely to related to both canopy structure (e.g., persistent needle cover increasing drag) and anchorage limitations of the roots of trees in relatively shallow urban soils. Our findings are aligned with the results of previous studies, as they observed that evergreen gymnosperms were uprooted more frequently during windstorms than other species [26]. Furthermore, softwood gymnosperm species (pine, spruce, fir, hemlock, cedar, redwood, and cypress) are more likely to be uprooted or have their trunks snapped at lower wind speeds than hardwood species such as oak, maple, birch, and ash [27,28].

Interestingly, tree species native to the Hungarian flora generally exhibited greater resistance to storm-induced wind damage than those that have been introduced to the region. This supports the notion that native species are generally more resilient to extreme weather events than species established outside their original home range [7]. In contrast, however, tree species native to the Debrecen region were more likely to suffer damage during the storms. This apparent “nativity paradox” may well reflect differences in provenance and local adaptation. It is plausible that species originating from slightly warmer or drier parts of Hungary are now better suited to Debrecen’s increasingly warm and hydrologically altered urban microclimate, characterised by the heat island effect, reduced soil permeability, and compacted substrates, than the historically local ecotypes.

In this sense, local native species may be maladapted to the novel conditions of the urban environment, whereas non-local but regionally native species may display a form of pre-adaptation to such stresses. This interpretation is consistent with the emerging framework of climate-adjusted provenancing, which advocates for the deliberate use of genetic material from climatically analogous, but not necessarily local sources to enhance resilience under climate change [29]. Similar reasoning underpins the concept of assisted migration, already operationalised in forest management and restoration contexts [30,31]. Extending these ideas to urban forestry suggests that adaptive selection of provenances, rather than strict reliance on local ecotypes, may improve the long-term stability and resilience of urban tree populations. This hypothesis warrants further investigation in the context of urban forest restoration and climate-sensitive planting strategies.

The spatial context also played a critical role in the likelihood of trees being damaged during the storms. The presence of nearby buildings, particularly taller structures, reduced the probability of trees being damaged, likely because they directly sheltered trees from the windstorms. Our findings corroborate previous studies that highlight the large influence of built environment configuration on micro-scale wind dynamics and the likelihood of trees being damaged during storms [8,15,28]. However, it is prudent to consider that excessive sheltering against wind is also likely to restrict tree growth or root development over time [8], thereby highlighting the need for a balanced approach to green infrastructure planning in urban areas.

Our findings also highlight species-specific variation in vulnerability to storms, with *Acer saccharinum*, *Platanus* × *hispanica*, and *Morus alba* among the most frequently uprooted tree species. Each of these tree species are commonly planted in urban environments in Hungary due to their tolerance of pollution and compacted soils in modified habitats. However, their structural fragility during extreme wind events raises serious questions about their long-term suitability as trees to plant in urban areas under future climatic conditions in which extreme weather events are predicted to occur ever more frequently.

### 3.1. Societal Perceptions and Adaptive Awareness

Beyond biophysical assessments of tree damage during extreme weather events, our study incorporated residents’ perceptions of storm frequency, causality, and mitigation measures, which is a rarely integrated yet essential aspect of the creation and management of urban trees [25]. A majority (70%) of participants perceived an increase in storm frequency over recent decades and further attributed such as an increase predominantly to climate change. This perception is well supported by studies showing that extreme weather events have increased in occurrence in central Europe over recent decades due to atmospheric instability and altered jet stream patterns [7].

Such high levels of public awareness of the climatic drivers of windstorms further suggest a developed societal sensitivity to global environmental changes. However, the occurrence of a diverse array of causal mechanisms, including environmental degradation, divine will, and even conspiracy theories, indicates a highly varied public understanding of the causes of damage because of extreme weather conditions [25]. This level of variation in opinions further highlights the importance of targeted science communication and community education endeavours to foster informed environmental engagement.

The most frequently proposed strategies to cope with extreme weather events focused on localised, symptomatic interventions (e.g., pruning, tree health monitoring), while systemic and climate-oriented responses (e.g., tree species selection, environmental policies, and lifestyle changes) were less frequently mentioned by the people we interviewed. This asymmetry reflects a knowledge-action gap because whilst people recognise the growing threat associated with climate change and extreme weather events, many feel disempowered to contribute meaningfully to mitigation against these threats. Indeed, 38% of respondents either expressed resignation or were unable to suggest any concrete action that could ameliorate the effects of windstorms. These findings underscore the need for participatory urban forestry strategies that not only address biophysical resilience but also enhance the citizens’ sense of agency and stewardship [25].

### 3.2. Implications for Urban Planning and Climate-Adaptive Forestry

The integration of ecological, structural, and social data in this study provides a basis for a holistic approach to climate-aware urban tree management globally, although they are particularly relevant for temperate regions. Our findings support several practical recommendations.

First, tree species selection when planting new trees should prioritise structurally resilient species that exhibit a strict tolerance of urban stressors and are adapted to withstand extreme weather conditions. While non-native trees are usually the most planted species in urban areas due to their ornamental value or pollution tolerance, our findings suggest that caution is required because species such as *Acer saccharinum* or *Morus alba* are prone to being damaged during extreme weather events and further suggests that greater structural assessment and site-specific justification are needed to plant trees that are resilient to high winds [2.28]. Similarly, evergreen gymnosperms, though visually appealing, may require careful placement and anchorage support in wind-exposed areas and so greater though regarding where to plant such trees is required.

Second, urban design must consider the protective and amplifying effects of the built environment, primarily in the form of buildings and other infrastructure, on wind dynamics at microgeographic scales. Accordingly, the strategic placement of trees in relation to buildings, road corridors, and open intersections such as parks can mitigate the impacts of strong winds and therefore reduce risk [2]. Our data further suggest that dense building cover may offer passive shelter from strong winds, but long-term planning commitments should also address sub-surface constraints (e.g., rooting volume, soil compaction) that strongly affect tree stability. In addition, developers, architects, urban planners, and builders should be encouraged to incorporate trees into the earliest stages of planning procedures and to include stabilising mechanisms (e.g., underground wires, structural soils) to prevent subsequent wind damage. City officials should review proposed plans to ensure that trees have been integrated from the outset and that appropriate stabilising measures or soils are provided. Moreover, these stabilisation mechanisms could also be applied to street trees that are not directly adjacent to buildings, further enhancing overall urban resilience to extreme wind events in the future.

Third, damage risk modelling, which is accompanied by fragility assessments, remote sensing, and longitudinal weather data, should be further developed. Regionally calibrated early-warning systems and decision-support tools can thereby assist municipal authorities in proactive risk management. Such management may take various forms but includes pre-storm pruning of branches likely to be torn from the main tree structure, emergency response planning, and targeted tree replacement strategies.

Finally, fostering climate literacy and civic participation is also essential to create an effective approach to dealing with threats to trees from extreme weather events [25]. Urban trees are not merely passive elements of green infrastructure, they are shared socio-ecological assets whose management requires public trust, awareness, and involvement. Collaborative platforms that integrate expert knowledge with local insight, such as citizen tree inventories, climate workshops, and participatory planning forums, may help bridge the gap between urban forestry science and public engagement to create urban landscapes whose trees are resilient to ongoing changes in climate and are also beneficial to humans.

Our study provides a framework for assessing storm-induced damages to urban trees, integrating fieldwork observations and satellite imagery with interviews of citizens. By enabling the establishment of broader, global databases, our approach paves the way for standardised monitoring tree damage and comparative analyses across regions and continents. Such unified data sources are critical not only for understanding the increasing vulnerability of urban trees in a changing climate but also for guiding urban planning, risk mitigation, and biodiversity conservation efforts. Ultimately, integrating storm damage assessment into urban ecosystem management will be essential for fostering more resilient, sustainable, and nature-inclusive cities.

### 3.3. Limitations and Future Research

Our study has several limitations that should be acknowledged and considered when drawing conclusions from our data.

First, although the statistical models accounted for site-level variability through random effects, we lacked detailed quantitative data on local tree management intensity or maintenance practices at each site. While the inclusion of a random intercept for location partially controls for these differences, unmeasured variation in maintenance quality may still have influenced the observed damage patterns. This means that more detailed information about tree management within study sites would have been useful in informing effects of microgeographic scales.

Second, wind-related variables were not available at the scale of individual trees. As storms are unpredictable and city-wide wind monitoring at tree-level resolution is not usually feasible, we could not incorporate local wind speed or gust intensity into the models. This limitation restricts our ability to fully disentangle the effects of wind exposure from the influence of tree and site-level characteristics. Such detailed measurements would be invaluable for future studies investigating storm-induced wind damage in urban landscapes.

Third, the social survey component relied on sampling participants who voluntarily engaged with the research team in the aftermath of the storms. This approach was useful but may have introduced bias, as individuals who experienced severe damage to their property or held strong opinions about tree safety could well have been more motivated to participate in our surveys. Consequently, public concern or perceived risk levels reported in our study may be overestimated when compared to a random subset of residents. To mitigate this limitation, we complemented the in-field interviews with an online questionnaire, which could be further developed and disseminated more extensively at national or international levels in future research and avoids a potential bias towards people who have been affected by storms.

## 4. Materials and Methods

### 4.1. Study Area

**Figure 4:**
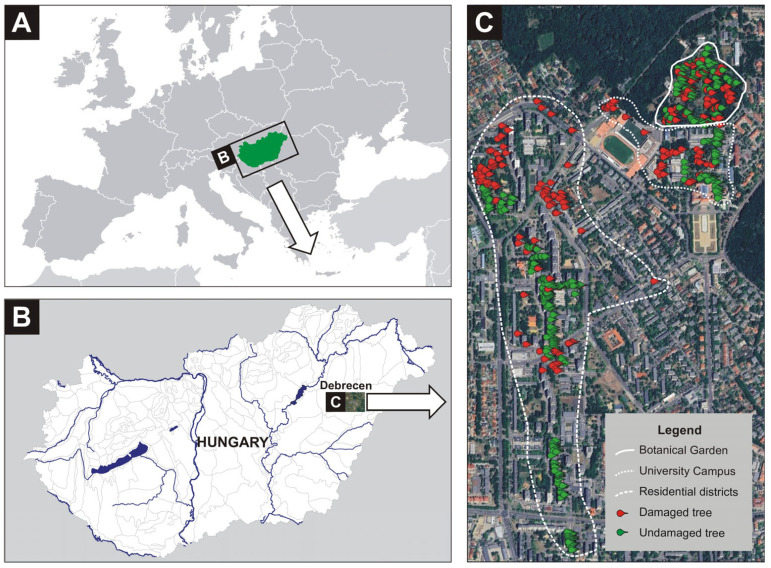
The location of Hungary within Europe (**A**), the location of Debrecen within Hungary (**B**), and the distribution of the surveyed trees (**C**).

This study was conducted within the administrative boundaries of the city of Debrecen, north-eastern Hungary (Figure 4). Geographically, the area lies within the Nyírség region, characterised by a continental climate and calcareous-free sandy soils. The natural vegetation of this phytogeographical zone is dominated by closed lowland steppic oak forest, with *Quercus robur* being the prevailing tree species [32].

Three distinct urban areas were surveyed: two residential districts (Újkert and Vénkert), which comprised 4- to 10-storey apartment buildings; the parkland surrounding the University of Debrecen; and the adjacent Botanical Garden. Although all the sites are embedded within urban areas, their vegetative characteristics differ substantially.

The University campus and Botanical Garden are in the Nagyerdő (“Great Forest”) area in the northern part of the city and were established during the 1910s and 1920s. Remnants of the original forest vegetation likely persist, as evidenced by the presence of old and massive *Quercus robur* trees. Other scattered native tree species of smaller stature, such as *Crataegus monogyna, Pyrus pyraster, Rhamnus cathartica*, and *Tilia cordata*, may also reflect relics of the original flora. The Botanical Garden, meanwhile, by its very nature, harbours a diverse range of ornamental and non-native plant species, including trees, and provides recreational opportunities for visitors.

In contrast, vegetation in the residential districts mainly consist of tree species that have been planted (*Acer platanoides*, *Tilia tomentosa*, *Acer saccharinum*, *Platanus × hispanica*, *Celtis occidentalis*) in urban green spaces for their tolerance to anthropogenic stress. Occasionally, fruit trees (*Prunus cerasifera*, *Pyrus communis*, *Juglans regia*) were also planted in the residential zones.

### 4.2. Field Data Collection

Fieldwork on damaged and undamaged trees was conducted between 8 and 15 July 2025, which is immediately after the two extreme windstorms occurred. The total area surveyed amounted to approximately 395,000 m^2^ in the two residential districts, 39,000 m^2^ on the University campus, and 81,000 m^2^ in the Botanical Garden.

A total of 201 damaged trees in the three areas were assessed for estimating damage. For each tree that was damaged, it was identified to the species level, and its geographic coordinates were recorded, and the circumference of their trunks at breast height (CBH) were measured using a tape measure. Damage to the trees was classified into one of the three categories: (1) trees with one or more broken branches that occurred during the preceding storms (referring to trees that sustained partial canopy damage due to branch falling), (2) trees with trunk breakage (referring to trees whose trunks fractured at or near mid-height, resulting in the loss of the entire canopy), and (3) uprooted trees (referring to trees that were completely overturned, with their root systems dislodged from the soil). To quantify the rate of damage, we photographed each tree to capture its entire canopy after wind damage. For the fallen branches, both the basal diameter (along the main axis) and total branch length were measured using a tape measure. To approximate the extent of canopy damage, we combined a visual assessment of the proportion of broken crown material found on the ground with direct measurements of the diameter and length of broken branches. Based on the photographs taken, we counted the number of branches of comparable size in the tree canopy. Using this information, two research team members independently estimated the extent of the damage to the trees. The same pair of team members conducted all damage estimations throughout the study, thereby reducing the possibility of between-observer variation impacting our results. The two estimates were then averaged. These measurements were used to estimate the volume of lost canopy relative to the estimated original (pre-storm) crown volume. In cases of trunk breakage or uprooting, the damage rate was assumed to be 100%.

To assess the population structure of trees and to contextualise the damage caused by the windstorms, data were also collected on 325 undamaged trees in the three surveyed areas so they could be compared with the damaged trees. For these undamaged trees, they were identified to the species level and their geographic coordinates, and CBH were recorded. Trees were identified to the species level using the most recent Hungarian flora guide [33], while nomenclature followed the TRY database [34].

### 4.3. Supplementary Data Processing

Tree height was estimated from photographs taken during the field surveys, using the known height of participants in our study as a reference marker to extrapolate from, and thereby calculate tree height. The immediate surroundings (within a 150-metre radius) of each tree were characterised using high-resolution aerial imagery via Google Earth Pro. For each tree, the distance to, and the height of nearby buildings, located in the direction of the prevailing wind (west or north-west) were quantified.

To assess the nativity status of trees, all recorded tree species were categorised based on their nativeness at two spatial scales: (1) within Hungary and (2) within the administrative boundaries of the city of Debrecen. A species was considered native if it occurs naturally (i.e., without human introduction or influence) within the region and introduced if its presence is the result of intentional or accidental human planting or dispersal. The classification was based on distribution maps provided by the Pannonian Database of Plant Traits (https://padapt.eu/) (accessed on 28 August 2025) [35], which distinguish between native and introduced plants, including trees, in the regions of interest.

### 4.4. Perceptions of Storm Frequency and the Causes of Storms

To assess the opinions of residents living in Debrecen on the frequency of storms and suggestions to avoid damage to their properties and parkland etc, in-person interviews were conducted following the storms in July 2025. Residents often approached researchers whilst they were performing fieldwork and expressed a keen interest in the study. Their spontaneous engagement and curiosity highlighted the relevance of the research topic to the public, particularly considering recent extreme weather events. We therefore performed semi-structured interviews with adults who approached fieldworkers in this manner, following the method advocated by Newing et al. [36]. In-person interviews were also performed in September 2025 (approximately two months after the storm) by randomly asking residents to participate in our study and complemented by an online survey via Google Forms in early October 2025. 71 interviews were carried out on the University campus, while 79 took place in the streets of Újkert and Vénkert districts, and an additional 54 online interviews were collected.

Participants were asked three open-ended questions:

1. How has the frequency of storms changed over the past few decades?
2. What are the causes of storms?
3. How can we protect ourselves from storms and mitigate their impacts?

Respondents were encouraged to answer freely and were not prompted to answer in a specific manner. During interviews, detailed notes were systematically recorded on pre-printed data sheets, and the age and gender of each participant was documented. Of those people interviewed or reached online, 93 (46%) were men and 111 (54%) were women, whilst the average age (mean ± SD) was 41.9 ± 20.6 years (range: 17 to 89 years).

Following the completion of interviews, the responses were categorised and coded. For instance, in response to the question “What are the causes of storms?”, answers such as “people”, “people and cars”, and “humankind” were all grouped under the category “Human impact”.

### 4.5. Statistical Analysis

To examine the factors influencing the level of storm-induced wind damage to urban trees, we applied a zero-inflated mixed-effect beta regression model implemented in the ‘glmmTMB’ package [37,38] in R v4.2.1. [39]. This approach allowed us to simultaneously model (Equation (1)) both the conditional mean (${µ}_{i}$) in the response variable ($Y_{i}$, which was the proportion of damage to each of the tree) and the probability of excess zeros ($\pi_{i}$).

(1) $$Y_{i}\sim \left\{\begin{array}{c} 0,\;with\;probability\;\pi_{i}\\ Beta({µ}_{i}\phi ,{(1-µ}_{i})\phi ),\;with\;probability\;1-\pi_{i}\; \end{array}\right.$$

We used the damage rate of trees as the response variable, where undamaged trees (*n* = 325) were assigned a value of 0, whilst trees that were uprooted or with trunk breakage were assigned a value of ~1 (0.99 due to the applicability of the beta regression model), and trees with broken branches being assigned a value between 0 and 1 (following the estimation process described above).

We included the systematic position (angiosperm or gymnosperm), nativity status (native or introduced) at both national and regional levels, trunk circumference, canopy height, distance to nearby building and the height of the nearby building as predictor variables ($x_{i}$) both in the conditional (Equation (2)) and zero-inflated (Equation (3)) component of the regression model. We did not estimate the height of buildings outside the 150-metre range, so we prepared two separate models with and without nearby building height to maximise sample sizes (*n* = 389 and 526, respectively). Both models included location (residential districts, University campus or Botanical Garden) as a random intercept ($b_{0,location[i]}$, $u_{0,location[i]}$) to account for the pseudo replication of data from trees within the same study sites, with there being variation in microclimates and management activities within study sites.

(2) $$logi\left({µ}_{i}\right)=\beta_{0}+\beta_{1}x_{1i}+\ldots +\beta_{n}x_{ni}+b_{0,location[i]}$$

(3) $$ogi\left(\pi_{i}\right)=\gamma_{0}+\gamma_{1}x_{1i}+\ldots +\gamma_{n}x_{ni}+u_{0,location[i]}$$

We performed a stepwise model selection procedure by eliminating the predictors with the highest *p* value (*p* > 0.05) to find the minimally adequate model with the best fit to the data. After 3 or 4 steps, we stopped the selection procedure when the step reached the lowest Akaike’s Information Criterion value corrected for small sample size (AICc), which thereby indicated the best-fitting model. We performed model diagnostics available in the ‘DHARMa’ package [40] to evaluate the fit of each model.

## Figures and Tables

**Table 1:** The number of undamaged and damaged trees recorded in the three study areas in the city of Debrecen, Hungary. Percentages shown in parentheses refer to the total number of trees surveyed in each study area.

	Residential Districts	University Campus	Botanical Garden
Total number of trees surveyed	277	80	169
Undamaged trees	170 (61.4%)	51 (63.8%)	104 (61.5%)
Damaged trees	107 (38.6%)	29 (36.2%)	65 (38.5%)
Trees with one or more major broken branches	95 (34.3%)	21 (26.3%)	47 (27.8%)
Trees with trunk breakage	5 (1.8%)	4 (5.0%)	4 (2.4%)
Uprooted trees	7 (2.5%)	4 (5.0%)	14 (8.3%)

**Table 2:** Tree species recorded during the surveys, including their systematic position (G—gymnosperm, A—angiosperm), native range, and native status in Hungary and in the city of Debrecen, as well as the number of damaged and undamaged individual trees in July 2025.

Species (Systematic Position)	Native Range	Native Status (Hungary)	Native Status (Debrecen)	Undamaged Trees	Damaged Trees
*Abies alba* Mill. (G)	Europe	Introduced	Introduced	–	1
*Abies cilicica* Hook. (G)	Mediterranean	Introduced	Introduced	4	4
*Abies nordmanniana* (Steven) Spach (G)	Caucasus	Introduced	Introduced	4	1
*Abies pinsapo* Boiss. (G)	Iberian Peninsula	Introduced	Introduced	2	1
*Acer campestre* L. (A)	Europe and W Asia	Native	Native	6	5
*Acer negundo* L. (A)	N America	Introduced	Introduced	2	5
*Acer platanoides* L. (A)	Europe and W Asia	Native	Introduced	37	21
*Acer pseudoplatanus* L. (A)	Europe	Native	Introduced	1	1
*Acer saccharinum* L. (A)	N America	Introduced	Introduced	8	23
*Aesculus hippocastanum* L. (A)	Balkans	Introduced	Introduced	6	–
*Ailanthus altissima* (Mill.) Swingle (A)	Asia	Introduced	Introduced	1	3
*Alnus glutinosa* (L.) Gaertn. (A)	Europe, W Asia and N Africa	Native	Native	1	–
*Betula pendula* Roth (A)	Eurasia	Native	Native	1	–
*Calocedrus decurrens* (Torr.) Florin (G)	N America	Introduced	Introduced	–	1
*Carpinus betulus* L. (A)	Europe	Native	Introduced	1	–
*Carpinus orientalis* Mill. (A)	SE Europe and W Asia	Native	Introduced	1	–
*Castanea sativa* Mill. (A)	S Europe and Asia Minor	Native	Introduced	1	1
*Catalpa bignonioides* Walter (A)	N America	Introduced	Introduced	2	2
*Cedrus atlantica* (Endl.) Manetti ex Carrière (G)	N Africa	Introduced	Introduced	–	1
*Celtis occidentalis* L. (A)	N America	Introduced	Introduced	24	5
*Chamaecyparis lawsoniana* (A. Murray) Parl. (G)	N America	Introduced	Introduced	2	–
*Corylus avellana* L. (A)	Europe, W Asia and N Africa	Native	Native	1	–
*Crataegus monogyna* Jacq. (A)	Europe, W Asia and N Africa	Native	Native	–	2
*Fagus sylvatica* L. (A)	Europe	Native	Introduced	–	1
*Fraxinus excelsior* L. (A)	Europe and W Asia	Native	Introduced	5	4
*Fraxinus ornus* L. (A)	Europe and SW Asia	Native	Introduced	10	–
*Fraxinus pennsylvanica* Marshall (A)	N America	Introduced	Introduced	3	–
*Gleditsia triacanthos* L. (A)	N America	Introduced	Introduced	8	2
*Hamamelis virginiana* L. (A)	N America	Introduced	Introduced	1	–
*Juglans regia* L. (A)	Eurasia	Introduced	Introduced	2	–
*Juniperus communis* L. (G)	Circumboreal	Native	Introduced	–	1
*Juniperus virginiana* L. (G)	N America	Introduced	Introduced	8	–
*Koelreuteria paniculata* Laxm. (A)	Asia	Introduced	Introduced	3	–
*Malus baccata* (L.) Borkh. (A)	Asia	Introduced	Introduced	1	–
*Malus domestica* Borkh. (A)	Cultivated, origin: Asia	Introduced	Introduced	1	–
*Morus alba* L. (A)	China	Introduced	Introduced	4	5
*Paulownia tomentosa* (Thunb.) Steud. (A)	China	Introduced	Introduced	–	3
*Phellodendron amurense* Rupr. (A)	Asia	Introduced	Introduced	2	2
*Picea abies* (L.) H. Karst. (G)	Europe	Introduced	Introduced	2	–
*Picea pungens* Engelm. (G)	N America	Introduced	Introduced	7	1
*Pinus nigra* J.F. Arnold (G)	Europe and W Asia	Introduced	Introduced	2	–
*Pinus ponderosa* Douglas ex C.Lawson (G)	N America	Introduced	Introduced	2	–
*Pinus sylvestris* L. (G)	Eurasia	Native	Introduced	3	2
*Pinus wallichiana* A.B.Jacks. (G)	Asia	Introduced	Introduced	1	1
*Platanus* × *hispanica* Mill. ex Münchh. (A)	cultivated hybrid	Introduced	Introduced	17	12
*Platycladus orientalis* (L.) Franco (G)	Asia	Introduced	Introduced	11	–
*Populus ×hybrida* Moench (A)	Cultivated hybrid	Introduced	Introduced	–	3
*Populus alba* L. (A)	Eurasia, N Africa	Native	Native	2	6
*Populus nigra* L. (A)	Europe, W Asia, N Africa	Native	Introduced	–	1
*Prunus cerasifera* Ehrh. (A)	Europe and W Asia	Introduced	Introduced	13	6
*Prunus serotina* Ehrh. (A)	N America	Introduced	Introduced	1	1
*Pseudotsuga menziesii* (Mirb.) Franco (G)	N America	Introduced	Introduced	4	2
*Pterocarya pterocarpa* Kunth (A)	Cultivated species, possibly Asia	Introduced	Introduced	3	–
*Pterocarya stenoptera* C. DC. (A)	China	Introduced	Introduced	–	1
*Pyrus betulifolia* Bunge (A)	Asia	Introduced	Introduced	1	1
*Pyrus communis* L. (A)	Europe and W Asia	Introduced	Introduced	1	1
*Pyrus pyraster* (L.) Burgsd. (A)	Europe and W Asia	Native	Native	1	1
*Quercus cerris* L. (A)	Europe and Asia Minor	Native	Introduced	3	–
*Quercus robur* L. (A)	Europe	Native	Native	25	14
*Quercus rubra* L. (A)	N America	Introduced	Introduced	1	1
*Rhamnus cathartica* L. (A)	Europe, NW Africa, W Asia	Native	Native	1	–
*Rhus typhina* L. (A)	N America	Introduced	Introduced	–	2
*Robinia pseudoacacia* L. (A)	N America	Introduced	Introduced	14	20
*Salix alba* L. (A)	Eurasia	Native	Native	–	1
*Sophora japonica* L. (A)	China	Introduced	Introduced	–	12
*Tetradium daniellii* (Benn.) T.G. Hartley (A)	E Asia	Introduced	Introduced	1	1
*Tilia cordata* Mill. (A)	Europe	Native	Native	10	–
*Tilia platyphyllos* Scop. (A)	Europe	Native	Introduced	1	1
*Tilia tomentosa* Moench (A)	SE Europe and W Asia	Native	Introduced	39	11
*Ulmus pumila* L. (A)	Asia	Introduced	Introduced	6	4

**Table 3:** The results of the minimally adequate zero-inflated mixed-effect beta regression models with damage rate to trees as the response variable and their predictors. Model 1 and Model 2 were evaluated when both excluding and including building height in the initial full models (see model description and steps of model selection in Section 4). The AICc values and their differences compared to the corresponding full model are shown below each model. “Not selected” indicates occasions when a predictor was eliminated from the final model during the model selection procedure. Significant results are marked with asterisks: * ≤ 0.05, ** ≤ 0.01, *** ≤ 0.001.

Predictors	Model 1				Model 2			
	β	SE	z	*p*	β	SE	z	*p*
Conditional part								
Systematic position (Angiosperm Vs. Gymnosperm)	1.29	0.32	4.08	<0.001 ***	2.02	0.67	3.02	0.003 **
Nativity status in Hungary (Introduced Vs. Native)	−0.51	0.21	−2.48	0.017 *	−0.39	0.23	−1.65	0.099
Nativity status in Debrecen (Introduced Vs. Native)	0.60	0.30	2.00	0.045 *	0.71	0.37	1.95	0.051
Breast height trunk circumference	−0.01	0.00	−2.94	0.003 **	−0.01	0.00	−2.78	0.006 **
Tree height	0.08	0.03	2.28	0.023 *	0.09	0.04	2.39	0.017 *
Building distance	Not selected				Not selected			
Building height	-				Not selected			
Zero-inflated part								
Systematic position (Angiosperm Vs. Gymnosperm)	1.01	0.36	2.78	0.006 **	1.61	0.79	2.03	0.042 *
Nativity status in Hungary (Introduced Vs. Native)	0.49	0.22	2.24	0.023 *	0.62	0.27	2.25	0.023 *
Nativity status in Debrecen (Introduced Vs. Native)	Not selected				Not selected			
Breast height trunk circumference	−0.01	0.00	−6.53	<0.001 ***	−0.01	0.00	−4.61	<0.001 ***
Tree height	Not selected				Not selected			
Building distance	−0.02	0.00	−6.35	<0.001 ***	−0.02	0.00	−6.11	<0.001 ***
Building height	-				0.08	0.01	5.68	<0.001 ***
AICc (Δ)	505.30 (5.83)				348.19 (3.86)			

## Data Availability

The original contributions presented in this study are included in the article/Supplementary Materials.

## Supplementary Materials

The following supporting information can be downloaded at https://www.mdpi.com/article/10.3390/plants14213366/s1, Table S1: Taxonomic identity, location and characteristics of the studied trees.

## Abbreviations

The following abbreviations are used in this paper:

CBH	Circumference at breast height
E	East
N	North
NW	Northwest
S	South
SE	Southeast
SW	Southwest
W	West
